# Ultrasensitive Gas Detection via Polarization-Mode Photothermal Interferometry in a Single-Mode Nanofiber Coupler

**DOI:** 10.1021/acs.nanolett.5c06094

**Published:** 2026-02-02

**Authors:** Pengcheng Zhao, Haihong Bao, Hoi Lut Ho, Shuangxiang Zhao, Wei Jin

**Affiliations:** † Photonics Research Institute, Department of Electrical and Electronic Engineering, 26680 The Hong Kong Polytechnic University, 999077 Hong Kong SAR, China; ‡ Photonics Research Center, The Hong Kong Polytechnic University Shenzhen Research Institute, Shenzhen 518057, China

**Keywords:** Optical nanofiber, Laser spectroscopy, Photothermal interferometry, Trace gas detection, Lab-on-fiber

## Abstract

Optical nanofibers (ONF) have emerged as versatile platforms for studying light-gas interactions at the micro/nanoscale, yet existing ONF gas sensors remain limited in detection sensitivity. Here, we report a polarization-mode photothermal interferometry technique that precisely measures the gas absorption-induced phase difference between two polarization states of the symmetric supermode of a single-mode ONF coupler. The high power density and large evanescent field associated with the ONF coupler enhance the efficiency of photothermal phase modulation, while the strong waveguide birefringence and noise-immune differential phase detection confer environmental immunity, jointly yielding an order-of-magnitude enhancement in the signal-to-noise ratio. With a 2 cm-long overcoupled ONF coupler, we achieved an acetylene detection limit of 6 ppb and an instability below ± 1.2% over 30 h. This compact ONF gas sensor, based on standard fused directional coupler technology, provides a promising route toward cost-effective and high-performance solutions for environmental monitoring and industrial applications.

## Graphical abstract

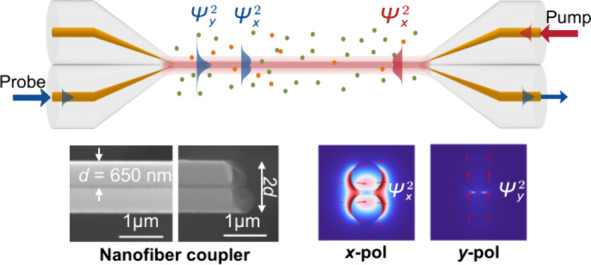

Gas sensors capable of detecting trace gases are essential for applications such as environmental monitoring and medical diagnostics.^1–3^ Optical methods, particularly laser absorption spectroscopy (LAS), are commonly used due to their high selectivity and sensitivity, leveraging light-gas interactions to measure the concentration of the absorbing gas.^4,5^ Among these techniques, pump–probe photothermal interferometry (PTI) has recently gained significant attention.^2,6–9^ In a typical PTI gas detection system, the pump light energy absorbed by gas molecules is converted into heat, known as the photothermal (PT) effect, which perturbs the refractive index (RI) of the gas medium.^10^ The resulting phase change of a probe light over the same optical path, which is related to the gas concentration, is detected via an optical interferometer. However, conventional PTI setups utilize complex and discrete optical components, which hinders miniaturization and field deployment, thus limiting practical applications.^11^

To overcome these challenges, PTI gas detection systems have been implemented in the form of fiber optics. The optical fiber systems offer several advantages such as remote sensing capability, electromagnetic immunity, and compact size, making them ideal for space-constrained or harsh environment applications.^12^ Optical nanofibers (ONFs), with subwavelength diameters, tightly confine light in modes with evanescent fields that interact strongly with the surrounding medium,^13^ enabling advances in optomechanical manipulations,^14–16^ quantum optics,^17–19^ nonlinear optics^20,21^ and optical sensing.^22–24^ An evanescent-wave PTI sensor has achieved a detection limit of sub-parts-per-million (ppm) level for acetylene (C_2_H_2_) gas by measuring the PT phase modulation with a two-beam Mach–Zehnder interferometer, where an adiabatically tapered fused silica ONF operating in the fundamental HE_11_ mode acts as the sensing arm.^25^ The high performance is attributed to the larger thermo-optic coefficient (TOC) and thermal expansion coefficient (TEC), as well as the higher peak light intensity around the ONFs compared to free-space beams and hollow-core fibers (HCFs),^3^ resulting in a larger effective RI modulation of optical modes. Higher-order modes (HOMs) could provide extra degrees of freedom to enable diverse mode field profiles for light-matter interaction,^26^ which has been successfully used in a mode-phase-difference (MPD) PTI that employs a two-mode (LP_01_ and LP_11_) HCF to detect the differential PT phase modulation between the two modes.^27^ The MPD is robust to ambient perturbations (e.g., temperature and pressure) as both modes propagate through the same hollow-core and are similarly affected by external disturbances. Common-path noise cancellation results in a noise-reduction factor ξ_nr_ on the order of *n* /Δ*n* = ∼10^2^, where *n* is the effective RI of the LP_01_ mode and Δ*n* is the RI difference between the LP_01_ and LP_11_ modes, which enables a low detection limit of tens of parts-per-trillion (ppt) for C_2_H_2_ gas. Mode evolution in a tapered optical fiber depends on the taper shape, and HOMs can be excited through a nonadiabatic process.^28,29^ Recently, evanescent-wave MPD-PTI has demonstrated enhanced detection sensitivity and stability by using a two-mode (HE_11_ and HE_12_, ξ_nr_ = ∼4) optical microfiber (OMF) interferometer for phase demodulation.^30,31^ Although the PT phase modulation efficiency in a tapered ONF/OMF can be over 10 times that in an HCF due to its excellent optical and thermal properties, the detection performance is still limited to the sub-ppm level, which remains insufficient for high-sensitivity gas sensing applications.

Here, we present an evanescent-wave polarization-mode PTI (PM–PTI) technique for gas detection with a single-mode bi-conical tapered ONF coupler. In addition to the spatial HOMs, polarization in optical fibers offers a complementary degree of freedom for tailoring light-matter interactions. Unlike conventional fused directional couplers, our ONF coupler has wavelength-scale dimensions and operates in an over-coupled state where it supports only the fundamental symmetric supermode (i.e., even mode) with two polarization states (or modes) while the fundamental antisymmetric supermode (i.e., odd mode) is cut off at both pump and probe wavelengths. The absorption-induced variation in the differential polarization mode phase is efficiently detected through a polarization-mode interferometer formed with the ONF coupler and a polarizer-analyzer pair. The differential phase measurement has inherent noise immunity and, when combined with the higher power density as well as the larger fractional evanescent mode power of the ONFs, enhances the signal-to-noise ratio (SNR) by an order of magnitude and achieves detection sensitivity at the parts-per-billion (ppb) level.

Figure 1a illustrates the fused ONF coupler-based evanescent-wave PM–PTI for gas detection. The 2 × 2 ONF coupler, tapered from two side-by-side standard single mode fibers (SMFs), has four input/output ports (port 1–4), a uniform waist and two adiabatic tapered regions. The waist region is formed by two side-contacting ONFs (SC-ONFs), each with a diameter *d* as shown in Figure 1b. The inset of Figure 1a presents scanning electron microscopy (SEM) images of the structure. The adiabatic tapered SC-ONFs can be regarded as a composite waveguide, and the fundamental even and odd modes can be excited simultaneously according to supermode theory.^32^ Figure 1c shows the effective RI of the fundamental even and odd modes as functions of diameter *d* at the wavelength of 1500 nm, calculated with COMSOL Multiphysics. For *d* between 0.32 and 0.7 μm, which is the diameter range of interest here, only the even mode can be excited due to odd-mode cutoff. Hence the SC-ONFs act as a single-mode waveguide with two orthogonal polarization states, whose electric field directions are along the minor (*x*-pol) and major axes (*y*-pol) of the dumbbell-shaped structure, respectively. Figure 1d presents the electric field (E-field) distributions of the *x*-pol and *y*-pol even modes at 1500 nm for the SC-ONFs with *d* ∼ 0.65 μm. Both the *x*-pol and *y*-pol even modes have large evanescent-field power fraction *Γ*_*m*_ (*m* = *x* or *y* for *x*-pol and *y*-pol), which is between ∼ 73% and ∼ 46% for diameter *d* from 0.6 to 0.7 μm, as shown in Figure 1c. The *y*-pol even mode mainly confines the evanescent field near the ONF contact area, while the *x*-pol even mode distributes the evanescent field around the ONF. *Γ*_*x*_ is slightly larger than *Γ*_*y*_. For reference, the E-field distributions of the odd mode at 1500 nm for *d* = 0.8 μm are also presented in Figure 1d.

**Figure 1:**
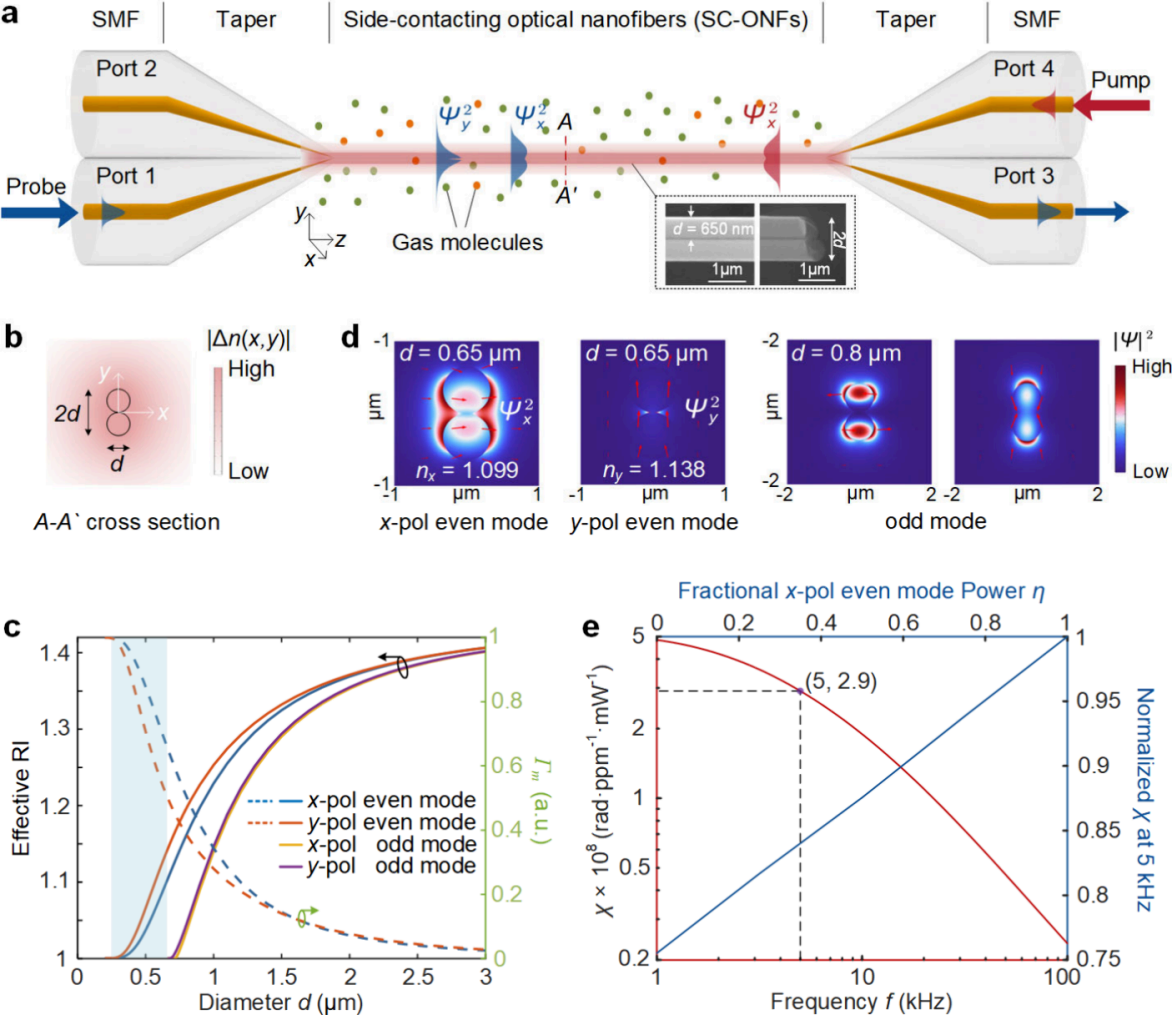
(a) Schematic diagram illustrating the PM–PTI technique for gas detection with an ONF coupler. The profiles of temperature (hence RI) perturbation in the SC-ONFs and surrounding gas medium are marked in light red. Inset: SEM images of the SC-ONFs in the *y*-*z* plane and a side view. (b) RI perturbation distribution over cross-section *A*–*A′* in (a) at 5 kHz. Two touching black circles indicate the geometry of SC-ONFs. (c) Effective RI of the even and odd modes of the SC-ONFs, and the evanescent-field power fraction *Γ*_m_ for the even mode as functions of the SC-ONFs diameter *d* at a wavelength of 1500 nm. The light-blue-shaded area indicates the diameter range of interest. The RIs of silica and surrounding air in the calculation are ∼1.444 and ∼1, respectively. (d) Cross-sectional E-field distributions of the even mode for *d* = 0.65 μm and the odd mode for *d* = 0.8 μm. The red arrows indicate the direction of the E-field. The *y*-pol even mode confines its evanescent field near the ONF contact surface, while the *x*-pol even mode distributes it around the ONF. (e) Calculated PMPD modulation coefficient *χ* as a function of modulation frequency *f* (Red line, bottom *x*-axis, left *y*-axis), and normalized *χ* as a function of the fractional *x*-pol pump power η at *f* = 5 kHz (Blue line, top *x*-axis, right *y*-axis).

The PM–PTI uses a pump–probe configuration, as shown in Figure 1a. Here we use C_2_H_2_ detection as an example, and the pump wavelength is selected to be ∼ 1532.83 nm that corresponds to the P(13) absorption line of C_2_H_2_. Linearly polarized (e.g., *x*-pol) pump light, modulated at frequency *f*, is launched into the SC-ONFs through port 4, which excites the *x*-pol even mode. Gas molecules absorb the pump light via evanescent field interaction, leading to local heating. This heating causes a change in the RI of the gas medium as well as the waveguide material via thermal conduction. The small-size geometry of the SC-ONFs, along with the higher thermal conductivity of silica compared to surrounding gases, makes the RI profile approximately uniform inside the fiber and gradually decaying outside, as shown in Figure 1b.

When a 45° linearly polarized probe light is launched into the SC-ONFs from port 1, it simultaneously excites the *x*-pol and *y*-pol even modes. The two polarization modes will undergo different phase modulation due to their different overlap with the RI distribution. Under weak absorption and negligible transmission losses, the polarization mode phase difference (PMPD) between the probe *x*-pol and *y*-pol even modes may be expressed as^30^

1 $$\Delta \varphi =\varphi_{x}-\varphi_{y}=\alpha \left(\lambda_{p}\right)CLP_{p}\cdot \chi \left(d,f,\eta \right)$$

where *φ*_*m*_ (*m* = *x* or *y*) represents the PT phase modulation for the probe *m*-pol even mode, α­(λ_p_) and *C* are respectively the absorption coefficient and the concentration of trace C_2_H_2_, *λ*_p_ and *P*_p_ are the pump wavelength and power, *L* is the length of the SC-ONFs. *χ* is a coefficient dependent on the ONF diameter *d*, the modulation frequency *f*, and the fractional *x*-pol pump power *η* (with the *y*-pol component being 1-*η*), which is determined by the polarization angle *θ* of the linearly polarized pump. The PMPD can be effectively detected by a polarization-mode interferometer, formed with a 45° polarizer at port 1 and a 45° analyzer at port 3.

Based on the numerical models in our previous works,^25,27,30^ we performed numerical simulations with the finite element method via COMSOL Multiphysics by considering the wavelength modulation technique with second harmonic (2*f*) detection (Note 1, Supporting Information). Figure 1e shows the computed PMPD modulation coefficient *χ* (left *y*-axis) as a function of modulation frequency *f* (bottom *x*-axis) for the fractional *x*-pol pump power *η* = 1 (i.e., all pump power is coupled into the *x*-pol even mode). The *χ* value decreases with increasing frequency, which is mainly governed by thermal dissipation. At *f* = 5 kHz, the calculated *χ* value is ∼ 2.9 × 10^–8^ rad·ppm^–1^·mW^–1^, which is ∼ 6 times larger than that in the 2.36-μm-diameter OMF used in our previous work.^30^ The enhancement arises because the evanescent-field peak power density of SC-ONFs is about one order of magnitude larger than that of the OMF, leading to a higher heat generation and thus RI modulation at a given pump power. Figure 1e also shows the normalized *χ* (right *y*-axis) as a function of the fractional pump power *η* in the *x*-pol even mode (top *x* -axis) at *f* = 5 kHz. The PMPD modulation coefficient *χ* with *y*-pol pumping is ∼ 76% of that with *x*-pol pumping, which means that the ideal configuration should couple all the pump power into the *x*-pol even mode (*η* = 1). This setup maximizes the pump evanescent field energy, which results in the strongest light-gas interaction and the largest PMPD modulation.

On the other hand, the PMPD exhibits much lower sensitivity to external perturbations compared with the phase of the individual fundamental even mode. This is because the extremely small diameter of the SC-ONFs ensures that environmental variations (e.g., temperature and pressure) induce nearly uniform RI changes around and inside the fiber, thereby influencing the phases of the *x*-pol and *y*-pol even modes in a similar way. Accordingly, the common-path noise cancellation factor may be given by^27^

2 $$\xi_{\mathrm{nr}}=n_{y}/\left(n_{y}-n_{x}\right)$$

where *n*_*x*_ and *n*_*y*_ are the RIs for the *x*-pol and *y*-pol even modes, respectively. For SC-ONFs with diameters *d* between 0.6 and 0.7 μm, the noise-cancellation factor *ξ*_nr_ is calculated to be ∼ 30, indicating much stronger noise suppression. These features enable a higher SNR, which can significantly improve the detection sensitivity with the evanescent-wave PM–PTI technique.

The experimental setup for gas detection follows a typical pump–probe photothermal spectroscopy configuration (Note 2, Supporting Information). The polarization-mode interferometer for phase detection is based on an ONF coupler, whose fabrication and characterization are briefly described in Note 3 of the Supporting Information. The SC-ONFs of the fabricated coupler have a length *L* of ∼2 cm and a diameter *d* of ∼0.65 μm. As shown in the inset of Figure 1a, the SC-ONF cross-section exhibits a dumbbell-like shape, consistent with ref 33. We first measured the frequency response and polarization-angle dependence of the PT signal by filling 1010-ppm of C_2_H_2_ in N_2_ into the gas chamber while tuning the pump wavelength to the P(13) line center. The amplitude of wavelength modulation voltage is set to ∼ 400 mV to maximize the 2*f* signal (Note 4, Supporting Information). Figure 2a shows the normalized 2*f* signal (blue dots) from the lock-in amplifier (LIA) when the wavelength modulation frequency *f* of the pump ranges from 500 Hz to 50 kHz. The PT signal decreases with increasing modulation frequency, consistent with the computed results (red line). The polarization-angle dependence of the PT signal was characterized at 6.64 kHz, which maximizes the system SNR (Note 4, Supporting Information). Figure 2b shows the normalized 2*f* signal (blue dots) when the polarization angle of the linearly polarized pump beam is changed from 0 to 180° with a rotating half-wave plate before entering the ONF coupler. The measurement results can be fitted with a squared sine function, as shown by the red line. The PT signal at a polarization angle of ∼0° or ∼180° (i.e., *y*-pol) is ∼75% of the maximum value obtained at ∼90° (i.e., *x*-pol), which is very close to the calculated value of ∼76% shown in Figure 1e. The discrepancy may be due to imperfect linear polarization of the generated pump beam, and different transmission losses between the two polarization modes. Figure 2c shows the 2*f* LIA output signals for different pump polarization angles when the pump wavelength is tuned across the P(13) line of C_2_H_2_. The gas sensing measurements in the following section were conducted at a polarization angle of ∼90° and a modulation frequency *f* of 3.32 kHz for the pump beam.

**Figure 2:**
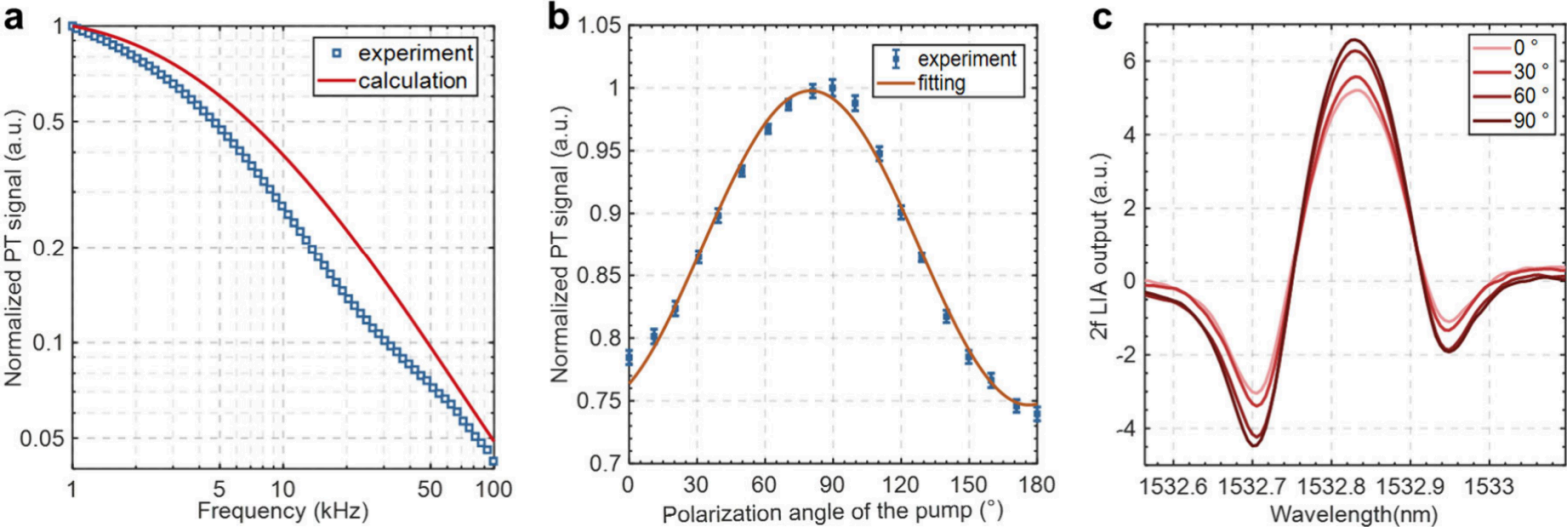
(a) Normalized PT signal at different demodulation frequencies. The calculated results correspond to the red line in Figure 1e. (b) Normalized PT signal as a function of polarization angle of the linearly polarized pump beam. Error bars in **b** show the standard deviation (s.d.) from 50 measurements. (c) 2*f* signals measured at different polarization angles of the linearly polarized pump beam.

We then evaluated the detection limit of the PM–PTI gas detection system. Figure 3a shows the *2f* LIA output signals at different pump power levels when the pump wavelength is tuned across the P(13) line of C_2_H_2_. Figure 3b presents the peak *2f* signal (PT signal) in Figure 3a and s.d. of noise (1σ noise) as functions of pump power. The baseline noise is recorded when the gas chamber is filled with pure N_2_ and the pump wavelength is fixed at the P(13) line center. The PT signal increases linearly with pump power with *R* ^2^ = 0.996, while the noise changes only slightly. With a pump power of ∼ 65 mW, the SNR for 1-s lock-in time constant is calculated to be ∼ 31141, giving a noise-equivalent-concentration (NEC) of ∼ 32 ppb C_2_H_2_. Allan–Werle deviation analysis was also conducted with the noise data collected over a 0.5-h period, and the results are shown in Figure 3c. The NEC goes down to ∼ 5.8 ppb C_2_H_2_ at an integration time of 252 s, corresponding to a noise equivalent absorption coefficient (NEA = *α·*NEC) of ∼ 6.1 × 10^–9^ cm^–1^.

**Figure 3:**
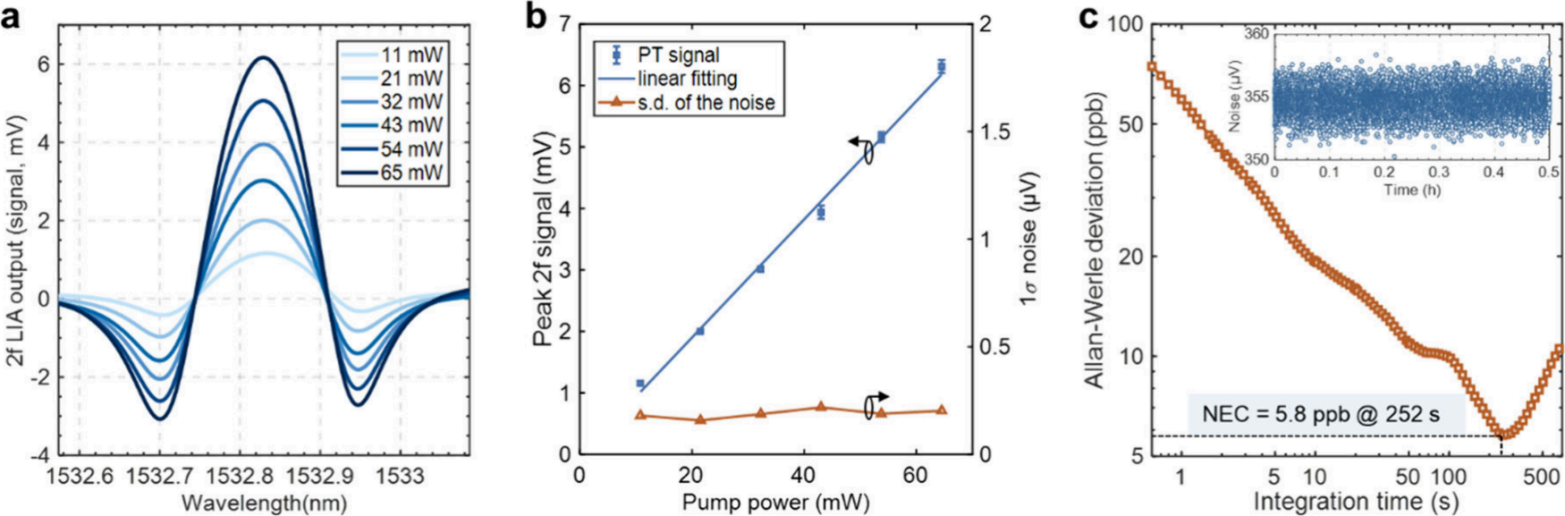
(a) 2*f* signals at different pump power levels. (b) Peak 2*f* signal and 1σ noise as functions of pump power. Error bars in (b) show the s.d. (×20) from 5 measurements. The lock-in time constant is 1 s and filter slope is 18 dB/Oct, corresponding to an equivalent noise bandwidth (ENBW) of 0.094 Hz. (c) Allan–Werle deviation plot based on noise data over a period of 0.5 h, as shown in the inset. The ENBW is set to 3.125 Hz.

The dynamic range (DR) of the system was evaluated by filling the gas chamber with different gas concentrations for 65 mW pump power (Note 5, Supporting Information). Figure 4a shows the *2f* LIA output signals of 100, 200, 350, 500, 780, and 1010 ppm of C_2_H_2_ at a flow rate of 101 standard cubic centimeters per minute (SCCM) at room temperature and atmospheric pressure. Figure 4b presents the PT signal as a function of gas concentration. A linear relationship can be fitted between the peak 2*f* signal and the C_2_H_2_ concentration over the range of 100–1010 ppm (R^2^ = 0.9999). The long-term stability of the gas detection system is tested over a period of 30 h in a lab environment. During the experiments, a constant flow of 1010-ppm of C_2_H_2_ at 5 SCCM was maintained to ensure a stable concentration inside the gas chamber. This flow rate was sufficiently low to avoid perturbing the PT signal. Figure 4c presents the continuous 1010-ppm of C_2_H_2_ measurement results, and the PT signal fluctuates within ± 1.2%, demonstrating a good long-term stability.

**Figure 4:**
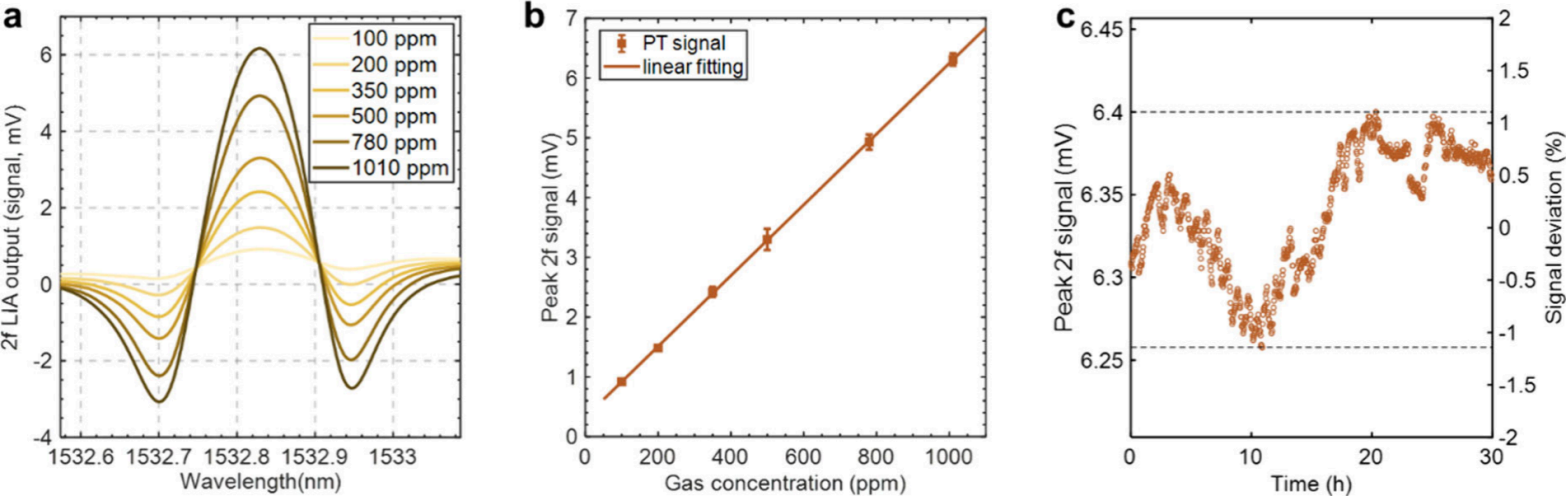
(a) 2*f* signals at different gas concentrations. (b) Peak 2*f* signal as a function of gas concentration. Error bars in (b) show the s.d. (×20) from 5 measurements. (c) Peak 2*f* signal variation over 30 h. Data in (a) and (c) were obtained with an ENBW of 0.094 Hz.

In conclusion, we report the first demonstration of a polarization-mode photothermal interferometry (PM–PTI) technique for trace gas detection with a compact single-mode ONF coupler. In a preliminary experiment, a detection limit of 6 ppb for acetylene gas was achieved, corresponding to a normalized noise-equivalent absorption coefficient (NNEA = NEA·*P*_p_·ENBW^–1/2^) of ∼ 6.5 × 10^–9^ cm^–1^·W·Hz^–1/2^. This performance surpasses previously reported ONF- and OMF-based spectroscopic techniques, as summarized in Table 1, and is comparable to the HCF-based approach^27^ when differences in fiber length are considered. Lower values of NEA, NEA·L, and NNEA indicate better detection performance.

**Table 1:** Performance of ONF- and OMF-Based Spectroscopic Gas Sensors^a^

Fiber type	Technique	NEA (cm^–1^)	NEA·L	NNEA (cm^–1^ W·Hz^–1/2^)	DR
**OMF**	MPD-PTI^30^	1.7 × 10^–7^	9.4 × 10^–7^	6.2 × 10^–8^	2.5 × 10^6^
**OMF**	MPD-PTI^31^	1.0 × 10^–6^	3.0 × 10^–7^	5.1 × 10^–7^	>1.0 × 10^4^
**OMF**	TDLAS^34^	1.4 × 10^–4^	2.8 × 10^–4^	N.A.	N.A.
**ONF**	MZI-PTI^25^	6.3 × 10^–7^	7.6 × 10^–7^	1.8 × 10^–7^	>1.1 × 10^3^
**ONF**	PAS^35^	1.5 × 10^–5^	N.A.	3.7 × 10^–6^	>1.5 × 10^3^
**ONF**	SI-PTI^36^	8.7 × 10^–8^	6.1 × 10^–8^	1.4 × 10^–8^	>3.8 × 10^2^
**ONF**	MPD-PTI^37^	3.5 × 10^–7^	2.1 × 10^–7^	5.0 × 10^–7^	N.A.
**ONF**	This work	6.1 × 10^–9^	1.2 × 10^–8^	6.5 × 10^–9^	>1.6 × 10^5^

a TDLAS, tunable diode laser absorption spectroscopy; MZI, Mach–Zehnder interferometer; PAS, photoacoustic spectroscopy; SI, Sagnac interferometer. The NNEA is a coefficient that is independent of gas types, absorption line strength, pump power, and detection bandwidth. NEA·*L* indicates the minimum detectable absorbance. N.A.: Not available.

The high performance of the PM–PTI technique arises from three main factors. First, precise control of adiabatic tapering enables operation in the over-coupling regime, where only the fundamental even mode is supported. The asymmetric dumbbell-shaped ONF coupler introduces strong birefringence and well-defined polarization axes, suppressing polarization mode coupling and improving environmental stability. Distinct mode field distributions lead to a large, measurable phase difference between the two polarization modes under absorption-induced RI modulation. Second, due to the very tight light confinement of the ONFs, both polarization modes exhibit significantly higher light intensity (over an order of magnitude greater than that of OMFs and considerably higher than HCFs) and larger fractional mode power in the evanescent field, which enhance the interaction between light and gas molecules to generate larger RI modulation. Finally, differential detection of the phase difference between polarization modes effectively isolates absorption-induced nonuniform RI modulation from external uniform disturbances (e.g., temperature and pressure), resulting in an order-of-magnitude enhancement in noise cancellation capability compared to previously reported ONF- and OMF-based systems. These features, combined with the larger TOC and TEC of silica material,^25^ enable improved SNR and eventually better detection performance.

Although the PM–PTI technique has demonstrated high detection sensitivity, further performance enhancement remains possible with higher-power pump sources,^38^ longer ONFs,^39^ and optimized gas pressure control.^40^ Notably, the ONF coupler is not essential for PM–PTI implementation and can be conveniently replaced with highly birefringent optical fibers, such as tapered micro/nanofibers^41,42^ and on-chip waveguides.^43,44^ Alternatively, the technique can also be applied to mid-infrared (MIR) spectroscopic gas detection by using MIR-transparent fibers,^45^ where gas absorption is significantly stronger than in the near-infrared. The response time is below 4 s (Note 6, Supporting Information), currently limited by the gas chamber volume rather than the PM–PTI mechanism itself, and could be reduced to less than 1 s by using a low-volume gas chamber with a sealed ONF coupler.^46^ Notably, the short fiber length, careful fixation, and high birefringence of the coupler effectively suppress polarization fluctuations, resulting in stable signals over 1 day. For extended operation, this issue could be further mitigated by employing polarization-maintaining fibers to minimize temperature- and stress-induced polarization coupling.

By addressing the key challenge of the limited detection sensitivity in ONF gas sensors, the PM–PTI technique opens new possibilities for designing small, cost-effective, and high-performance fiber-optic gas sensors. The compatibility of the ONF coupler with standard fused directional coupler packaging technology further makes it an attractive option for integration into existing optical fiber systems, paving the way for the development of next-generation ONF-based sensors (e.g., gas, liquid, chemical and biomarker) capable of meeting the demands of advanced sensing applications.

## Supplementary Material



## Notes

Numerical simulations for χ determination, experimental setup for gas experiments, fabrication and characterization of SC-ONFs, optimization of wavelength modulation technique, preparation of gas samples, response time measurement (PDF)
