# Supplementary material for: Ultrasensitive Gas Detection via Polarization-Mode Photothermal Interferometry in a Single-Mode Nanofiber Coupler
Source: Nano Lett. 2026 Feb 2;26(6):2249–55. doi: 10.1021/acs.nanolett.5c06094 (PMC12922175; doi:10.1021/acs.nanolett.5c06094)
Supplement: Supplementary file 1 [file nl5c06094_si_001.pdf]

Supporting information for

Ultrasensitive Gas Detection via Polarization-Mode Photothermal Interferometry in a Single-Mode Nanofiber Coupler

Pengcheng Zhao,[1,2,*] Haihong Bao,[1,2] Hoi Lut Ho,[1,2] Shuangxiang Zhao,[1,2] and Wei Jin[1,2,*]

[1] Photonics Research Institute, Department of Electrical and Electronic Engineering, The Hong Kong Polytechnic University, Hong Kong SAR 999077, China.

[2] Photonics Research Center, The Hong Kong Polytechnic University Shenzhen Research Institute, Shenzhen 518057, China.

[*]Email: zhaopc@buaa.edu.cn (P.Z.); eewjin@polyu.edu.hk (W.J.)

Supplementary note 1: Numerical simulations for χ determination

Based on the numerical models in our previous works [0(2)], we performed numerical simulations with the finite element method via COMSOL Multiphysics by considering the wavelength modulation technique with second harmonic (2*f*) detection. Numerical simulations were conducted using a pump power of 100 mW, and 1000 ppm C_2H_2 balanced with N_2. The pump and probe wavelengths are 1533 and 1570 nm, respectively. The absorption coefficient α of the P(13) line of C_2H_2 depends on pressure-induced line broadening, and is ~1.05 cm^{-1} for trace concentrations that only considers air broadening. The SC-ONFs have a diameter d = 0.65 μm, and parameters (density, thermal conductivity, heat capacity at constant pressure, and TOC) of materials used in the calculations could be found in **Table S1**.

Table S1. Parameters of materials at room temperature and atmospheric pressure

Parameter	Silica	Nitrogen
Density ρ [kg/m^3]	2203	1.16
Thermal conductivity κ [W/(m·K)]	1.38	0.0256
Heat capacity at constant pressure C_p [J/(kg*K)]	703	1040
Thermo-optic coefficient dn/dT [K^{-1}]	9.5×10^{-6}	-9.5×10^{-7}

Supplementary note 2: Experimental setup for gas experiments

Figure S1a shows the experimental setup for gas detection. The polarization modal interferometer for phase detection is based on the optical nanofiber (ONF) coupler. Figure S1b shows the transmission spectra of the ONF coupler, measured by a broadband source and an optical spectrum analyzer with a resolution of 100 pm. The red and green lines represent the measurement results from port 1 to port 3 and from port 1 to port 4, respectively, without a polarizer-analyzer (P2-P3 or P2-P1) pair (Thorlabs, FBR-LPNIR). At wavelengths below 1500 nm, optical power oscillates back and forth due to modal interference between the even and odd modes in the coupling region. At wavelengths beyond 1500 nm, the mode beating stops as one of the modes (i.e., odd mode) is cut off, which results in the side-contacting ONFs (SC-ONFs) supporting only the even mode above this wavelength threshold. According to the simulated results shown in Figure 1c, the cut-off diameter for the odd mode is ~0.7 μm at 1500 nm, which is very close to the measured value of ~0.65 μm. The blue lines represent the measurement result from port 1 to port 3, with a polarizer-analyzer (P2-P3) pair. The light beam passes through polarizer P2, generating both x- and y-pol modes, and is coupled out from port 3 of the ONF coupler. Analyzer P3 then recombines the two polarization modes to form a polarization-mode interferometer for polarization mode phase difference (PMPD) demodulation. To achieve the maximum fringe contrast of the interferometer, 45° linearly polarization is preferred for P2 and P3, which results in a fringe contrast of over 20 dB.

The pump beam is generated by a ~1533 nm seed DFB laser (FITEL, FRL15DCWD-A81-19560-C), and amplified with an erbium-doped fiber amplifier (EDFA) from Amonics (AEDFA-EX-27-B-FA). Its polarization state is adjusted with a polarization controller (PC1) and a rotating linear polarizer (P1) before being input into the SC-ONFs through port 4, and observed at port 2 with a commercial polarimeter (Thorlabs, PAX1000IR2). Wavelength modulation in combination with second harmonic ($2f$) detection is used in the experiment, where the pump wavelength is sinusoidally modulated at a high frequency of f to produce PT phase modulation. During polarization-angle-dependent measurements of the PT signal, a rotatable half-wave plate is inserted between P1 and Port 4 to vary the pump polarization and is removed afterward. The probe beam is from a tunable laser source (TLS) from Santec (TSL-570) with a wavelength range of 1500 to 1630 nm. The polarization state of the probe light is adjusted by using PC2 and P2 in the same way as for the pump light, and then launched into SC-ONFs. The probe beam coming out of ONF coupler is then passes through P3 and a

wavelength-division multiplexer (WDM), which filters out the residual pump beam, and is detected by two photodetectors (PDs). The DC component of the probe beam detected by the PD (New Focus, MODEL 2053) is used to stabilize the interferometer at quadrature (~1570 nm), indicated as the red dot in Figure S1b. The probe wavelength λ_{probe} is chosen near the dispersion turning point λ_{DTP}, as indicated by the black dot in Figure S1b, to enhance detection sensitivity [31]. Finally, the probe beam is detected by a balanced photodetector (BPD) from New Focus (MODEL 2117) to minimize the impact of probe laser intensity noise. A lock-in amplifier (LIA) from Zurich Instruments (MFLI 500k) is used to measure the 2*f* signal. The outputs from the PD and LIA were recorded by using a data acquisition (DAQ) card from National Instruments (USB-6001) and input into a computer.

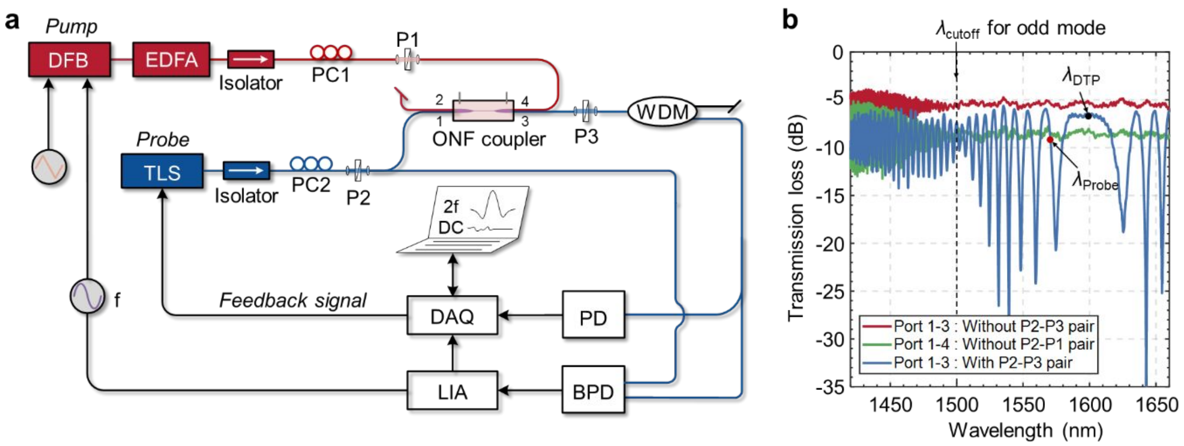

Figure S1. a. Experimental setup for gas detection using an ONF coupler with a 2-cm-long 0.65-µm-diameter SC-ONFs. DFB, distributed feedback laser; EDFA, erbium-doped fiber amplifier; PC1 and PC2, polarization controllers; P1-P3, rotating linear polarizers; WDM, wavelength-division multiplexer; TSL, tunable semiconductor laser; LIA, lock-in amplifier; PD, photodetector; BPD, balanced photodetector; DAQ, data acquisition card. **b.** Transmission spectra of the ONF coupler ranging from 1420 to 1660 nm with a wavelength resolution of 100 pm. The red and green lines represent measurements taken without any polarizer and analyzer, while the blue lines show measurements obtained by using the polarizer-analyzer (P2-P3) pair.

Supplementary note 3: Fabrication and characterization of ONF coupler

The ONF coupler was fabricated by tapering two twisted single-mode fibers (SMFs) with coating removed. The twisted region was stretched and fused together using the flame-brush technique, yielding SC-ONFs with a length of ~2 cm and a diameter of ~0.65 μm, with a diameter deviation within 5%. The insertion losses of the SC-ONFs from the port 1 to port 3 and port 4 are ~5.3 dB and ~8.5 dB, respectively. For gas experiments, the ONF coupler was mounted in a 12 × 1.4 × 1.5 cm^3 acrylic chamber.

Supplementary note 4: Optimization of wavelength modulation technique

The modulation amplitude and frequency were optimized by varying the modulation voltage and frequency applied to the laser controller of the pump source. The corresponding results are shown in **Figure S2a** and S2b, respectively. The wavelength modulation amplitude was set to approximately 400 mV to maximize the 2f signal, while the modulation frequency of ~6.64 kHz was chosen to maximize the signal-to-noise ratio of the gas detection system.

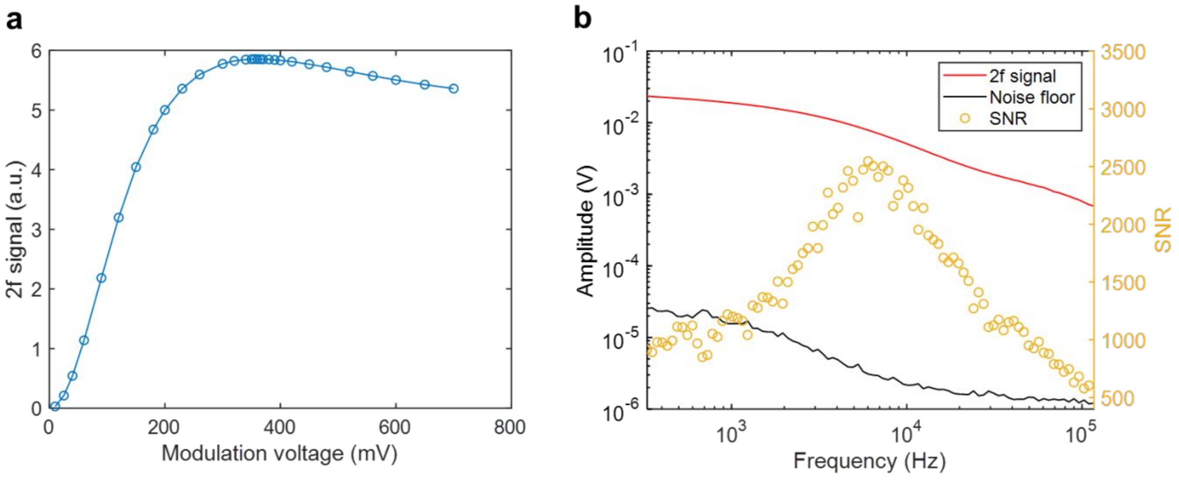

Figure S2. a. 2f signal as functions of wavelength modulation voltage of the pump beam. **b.** 2f signal as functions of modulation frequency of the pump beam.

Supplementary note 5: Preparation of gas samples

Gas samples with different concentrations were prepared at atmospheric pressure using two mass flow controllers. For concentrations below 1010 ppm, acetylene–nitrogen mixtures (1010 ppm C_2H_2 in N_2) were further diluted with high-purity nitrogen at different mixing ratios.

Supplementary note 6: Response time measurement

The sensor response time was characterized by sequentially filling pure N_2, 1010 ppm C_2H_2, and pure N_2 into the gas chamber at a flow rate of 200 SCCM, with the pump wavelength fixed at the center of the P(13) line. **Figure S3** presents the real-time 2*f* signal, baseline-corrected and normalized to its peak value. The response time, defined as the time required for the signal to reach 90% of its maximum value (rise time) and decay to 10% (fall time), was measured to be less than 4 seconds.

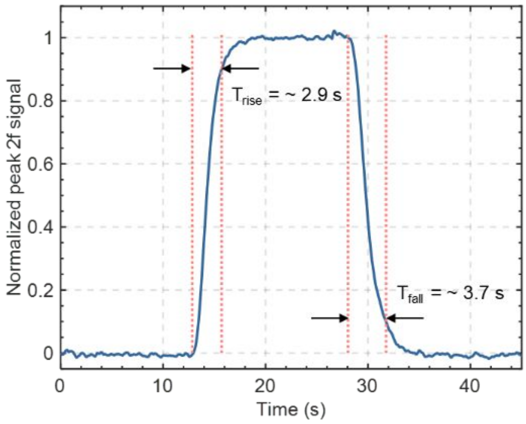

Figure S3. Response time measurement results. The ENBW is set to 3.125 Hz.

Reference

(1) Qi, Y.; Yang, F.; Lin, Y.; Jin, W.; Ho, H. L. Nanowaveguide enhanced photothermal interferometry spectroscopy. *J. Lightw. Technol.* **2017**, *35* (24), 5267–5275.

(2) Zhao, P.; Zhao, Y.; Bao, H.; Ho, H. L.; Jin, W.; Fan, S.; Gao, S.; Wang, Y.; Wang, P. Mode-phase-difference photothermal spectroscopy for gas detection with an anti-resonant hollow-core optical fiber. *Nat. Commun.* **2020**, *11*, 847.

(3) Zhao, P.; Zhao, Y.; Ho, H. L.; Jin W. Evanescent wave lab-on-fiber for high sensitivity gas spectroscopy with wide dynamic range and long-term stability. *Laser Photonics Rev.* **2023**, *17*, 2200972.
